# Personality traits influence contest outcome, and vice versa, in a territorial butterfly

**DOI:** 10.1038/s41598-019-39155-9

**Published:** 2019-02-26

**Authors:** Aurélien Kaiser, Thomas Merckx, Hans Van Dyck

**Affiliations:** Behavioural Ecology and Conservation Group, Biodiversity Research Centre, Earth and Life Institute, UCLouvain, Louvain-la-Neuve, Belgium

## Abstract

Holding a territory is often crucial in order to acquire key resources, including mating partners. However, few studies have investigated the role of animal personality in the context of territorial conflicts and how the contest outcome itself may influence personality traits. We studied personality in male Speckled wood butterflies, *Pararge aegeria*, before and after territorial contests for sunspot territories. Before interactions, boldness decreased with age, while activity and exploration were only influenced by ambient conditions. Neither age nor morphology did influence the probability to win contests, but winners were more active and more explorative than losers and, moreover, males that received a red wing mark were more likely to be winners. Butterflies that lost a contest showed pronounced behavioural changes. Mean boldness increased and its repeatability was disrupted, while no such change was detected in winners. The observed boldness increase in losers may be explained by a ‘desperado effect’, though its implication for successive contests remains unknown. Given that territoriality is expected to have important consequences for reproductive success, our results suggest that personality traits may indirectly contribute to individual fitness by influencing the ability to gain access to mate-location patches.

## Introduction

Resource acquisition often results in conflicts among conspecifics and animals may use auditory, visual or olfactory cues to deter potential competitors. However, the use of such signals is not always sufficient to settle conflicts and aggressive interactions may hence occur to exclude competitors from the resource. Contests are direct interactions that involve two or more individuals trying to gain access to a resource. They typically end with all but one of the opponents withdrawing from this contest or fight^1^. The ability to persist in a contest is called ‘resource-holding potential’ (RHP)^2^. Typical RHP traits include morphological traits –such as a large body size or weaponry– as well as physiological traits like endurance or motivation^3^. Contests, however, incur costs in the form of injuries and energy expenses. Additionally, a contest outcome may affect one’s ability to win subsequent contests. Individuals commonly have a higher chance of winning after a previous winning experience, whereas losers are less likely to win the next contest^4^. The duration of such winner and loser effects varies from a few hours (for example in some invertebrates^5,6^) to several days (e.g.^7,8^). Additionally, they may spill over into other behaviours. For instance, male *Gryllus bimaculatus* crickets that experienced repeated defeats reduced their mobility and avoided conspecifics later on^9^, while winner and loser effects depended on behavioural type in the Rainbow trout *Onchorhyncus mykiss*^10^.

Animal personality refers to individual behavioural differences that are consistent across time and/or contexts^11^. So-called personality traits have been reported from a wide range of taxa^12^ and often co-vary with each other, thus forming behavioural syndromes^13^. Additionally, they are linked to important eco-evolutionary processes^14^ and they influence individual fitness components such as survival^15–17^ and reproductive success^18–20^. Briffa *et al*.^21^ reviewed recent evidence for the role of personality traits on contest outcome. Although the number of studies is relatively small, the authors suggest that personality traits like boldness (i.e. an individual’s reaction to any risky situation^11^) and aggressiveness can contribute to RHP. Additionally, few studies highlighted post- contest changes in personality traits. For instance, boldness decreased following defeat in the sea anemone *Actinia equina*^22^, whereas post-contest changes in boldness in European hermit crabs (*Pagurus bernhardus*) were more complex; they depended on both the role of the focal individual (i.e. attacker or defender) and the contest outcome^23^.

In butterflies, male mate-locating behaviour can be broadly classified into two distinct strategies: patrolling and perching^24^. In some species, only one strategy is predominant, while in others both perchers and patrollers can coexist, and this even within a single population. Patrolling males fly almost continuously in search of mates, stopping only during short periods for basking or feeding. Contrastingly, perching males typically sit on a prominent site or object and await females^25^. When a passing object of appropriate size and colour is detected nearby, these perching males will fly directly towards it for inspection. Encounters with conspecific males usually result in a brief chase or in escalated contests. The latter typically involve aerial interactions between the opponents in the form of mid-air circling manoeuvres and end with one butterfly giving up the contest^26^. Perching males often do control a small area which they defend against intruders. As such, traits that influence the ability to persist in an aerial circling phase, and hence the ability to remain in control of this ‘territory’, can be seen as RHP traits^27^. In butterflies, contests have been widely studied and outcomes have been shown to depend on both morphological and physiological traits such as body size and age, although the magnitude to which these traits influence contest outcomes seems to vary among species (see^28^ and references therein).

The existence of consistent individual differences in behaviour (i.e. personality traits) has recently been shown for some butterfly species^29–31^. Yet, we are not aware of any study addressing the influence of personality traits on butterfly contests. Here, we studied how boldness, exploration and activity affect contest outcome in males of the Speckled wood butterfly (*Pararge aegeria*), while controlling for morphology and age. We expect bold and more active individuals to have a higher probability to monopolize a territory as active and risk-taking individuals may have an advantage during territorial contests^21^. However, we expect neither morphological traits (including body size) nor age to be significant predictors of contest outcome in this species^32–34^. We also analysed how contest outcome influences boldness. We expect winners and losers to display increased and decreased boldness, respectively, as a result of winner and loser effects^21^. Finally, our design also allowed us to explore age-related changes in personality traits. Because bold individuals may suffer from increased predation risk^35^, and because young animals have higher future fitness expectations than older conspecifics, we predict young individuals to show reduced risk-taking behavior^36^.

## Results

### Repeatability and correlation among behavioural traits

Of the three behavioural traits considered before the male-male contests, only boldness was repeatable (Table 1). The repeatability for this trait was moderate (0.399), whereas exploration and activity had repeatability estimates close to zero.

**Table 1:** Adjusted repeatability of boldness, exploration and activity, with their associated P-values.

Behavioural trait	Adj. repeatability	P-value
*Before contest*		
Boldness	**0.399**	**<0.0001**
Exploration	0.048	0.32
Activity	0.096	0.15
*Before-after contest*		
Boldness	Winners: **0.283**	**0.028**
	Losers: 0.007	0.48

Significant repeatability estimates and P-values are indicated in bold.

In both the first and the second trials, boldness was unrelated to activity (first trial: r = −0.06, df = 103, P = 0.512; second trial: r = 0.04, df = 104, P = 0.672). Similarly, boldness and exploration did not correlate (first trial: r = 0.09, df = 103, P = 0.312; second trial: r = 0.02, df = 104, P = 0.831). Activity and exploration were positively related in both trials (first trial: r = 0.52, df = 104, P < 0.001; second trial: r = 0.50, df = 104, P < 0.001).

### Factors influencing behavioural traits

**Figure 1:**
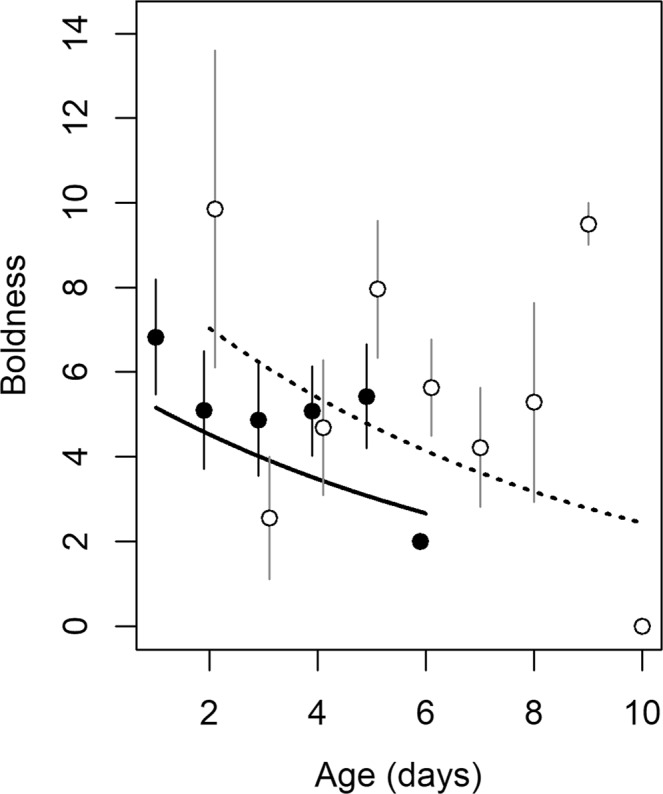
Effect of age and testing sequence on boldness (N = 105 and 106 for the first and second trial, respectively). Circles show observed age- and trial-specific boldness (mean ± SE) and lines represent predicted values based on the final model. Filled circles and the solid line refer to the first trial; open circles and the dashed line to the second trial. Points were slightly jittered with regard to age. The two points for which standard error is not provided are those with N = 1.

Boldness decreased with increasing age at testing (${{\rm{\chi }}}_{1}^{2}$ = 6.86; P = 0.008), and butterflies behaved bolder during the second test (${{\rm{\chi }}}_{1}^{2}$ = 6.34; P = 0.01) (Fig. 1). Fresh body mass had no impact on boldness (${{\rm{\chi }}}_{1}^{2}$ = 1.15; P = 0.28). Exploration and activity were influenced by environmental conditions: exploration decreased with increasing light intensity (${{\rm{\chi }}}_{1}^{2}$ = 6.68; P = 0.01), and tended to increase with increasing temperature (${{\rm{\chi }}}_{1}^{2}$ = 3.66; P = 0.055). Similarly, activity increased with increasing temperature (F_1,202.8_ = 11.78; P < 0.001), and there was a tendency for lower activity with increasing light intensity (F_1,199.9_ = 3.86; P = 0.051). We found no significant effect of age, trial number and fresh body mass on exploration and activity (all P-values > 0. 08).

### Predictors of contest outcome

**Figure 2:**
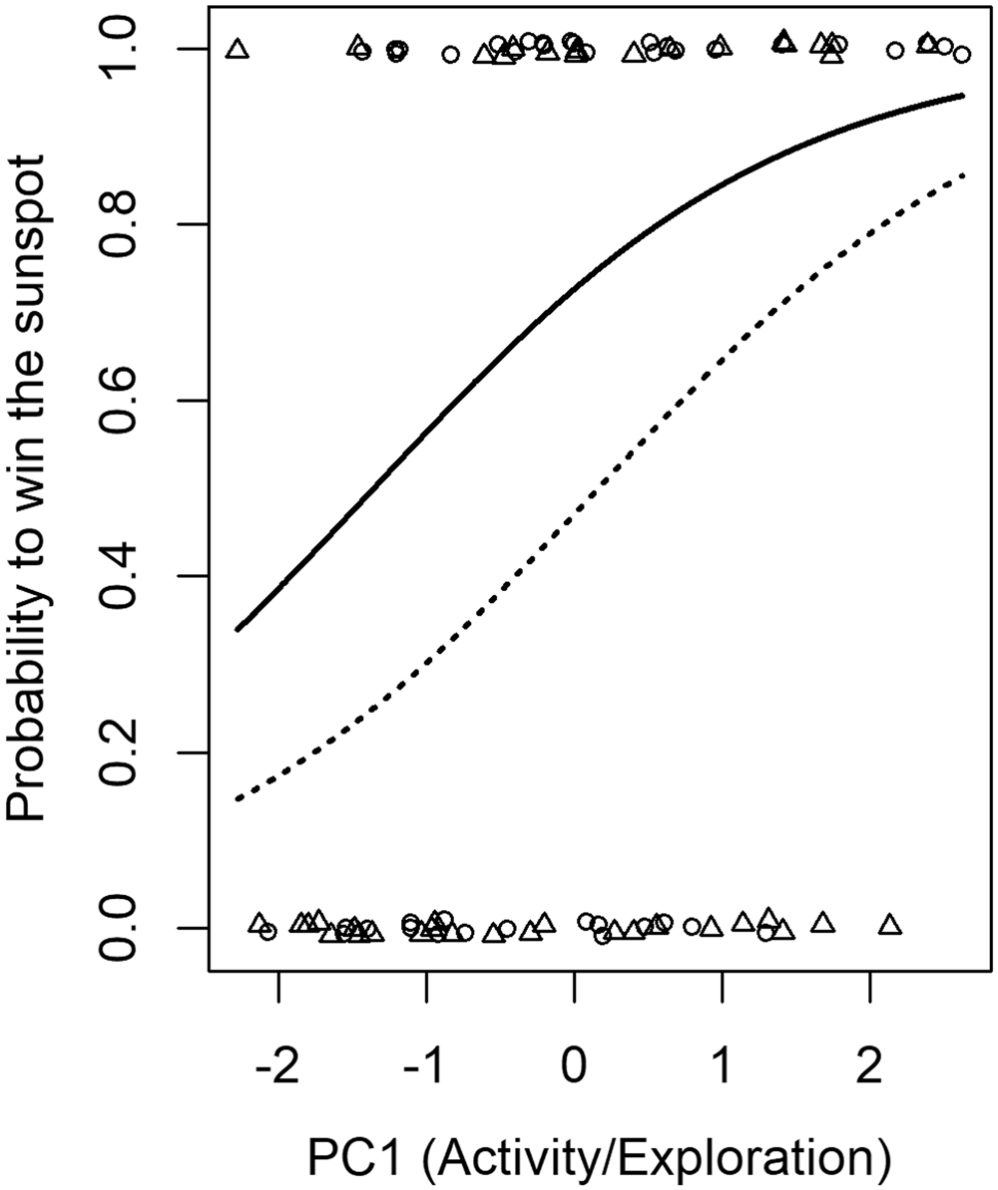
Probability to win the central sunspot as a function of PC1 and marking colour (red-marked individuals: circles and the solid line; green-marked individuals: triangles and the dotted line). Lines represent predicted probability based on the final model accounting for age, morphological traits and other behavioural PCs. Points were slightly jittered vertically in order to improve clarity.

Individuals with higher values of PC1 (i.e. with higher exploration/activity scores before the contest) and with red wing marks (compared to green wing marks) had a higher probability to settle in the sunspot and to win territorial contests (${{\rm{\chi }}}_{1}^{2}$ = 9.65; P = 0.018 and ${{\rm{\chi }}}_{1}^{2}$ = 4.09; P = 0.042, respectively) (Fig. 2). The other PC axes, age, aspect ratio and thorax ratio had no significant effect on contest outcome (all P-values > 0.08). Only forewing area (used as a proxy for body size) tended to influence the contest outcome (${{\rm{\chi }}}_{1}^{2}$ = 3.48; P = 0.062) with small individuals tending to have a higher chance to be winners.

### Effects of contest outcome on boldness

**Figure 3:**
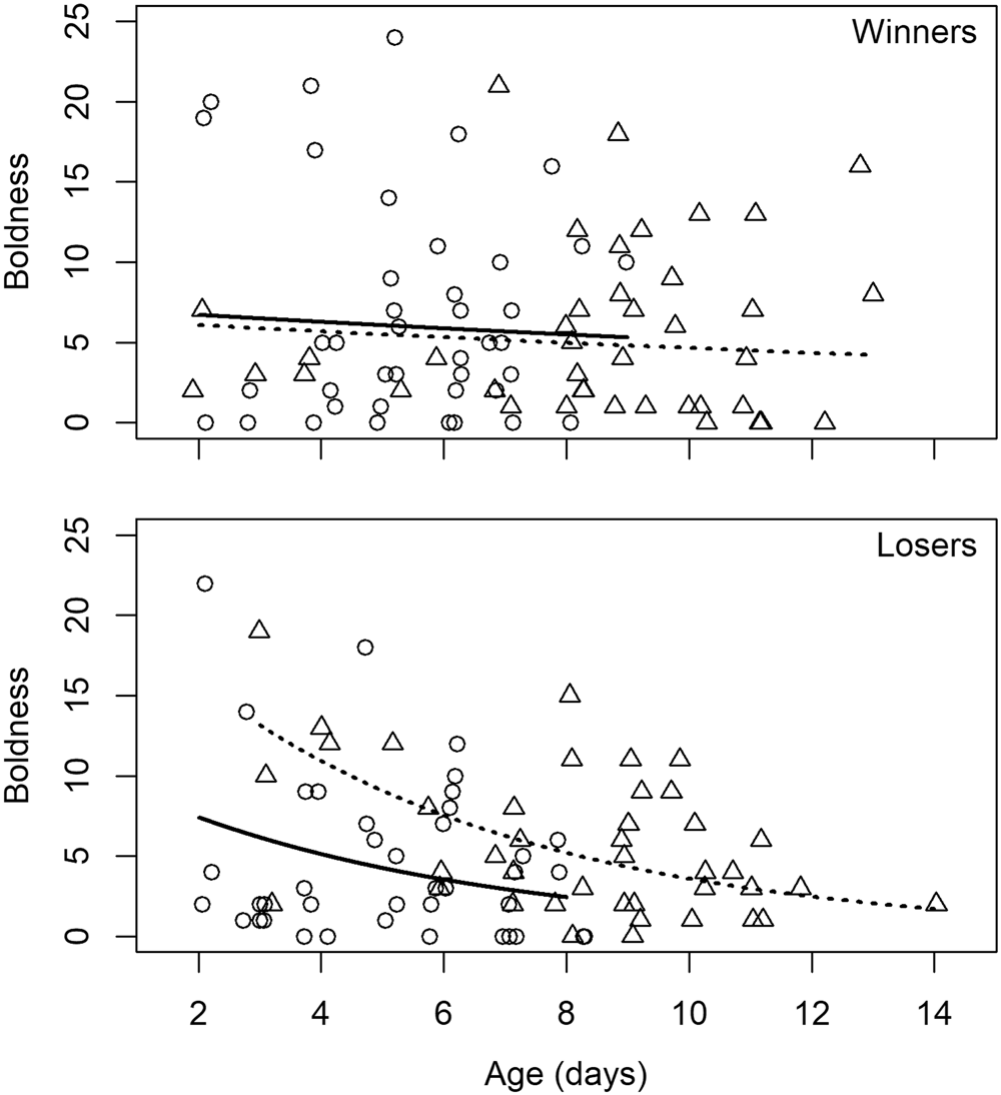
Effect of age and period (pre- *versus* post- contest) on boldness for contest winners (top) and losers (bottom). Circles and triangles show observed boldness for pre- and post- contest periods, respectively. Solid (i.e. pre- contest period) and dotted (i.e. post- contest period) lines represent predicted values based on the final model. Points are slightly jittered to improve clarity.

When comparing boldness before and after the male-male contest (i.e. pre- and post-contest periods), we detected significant age × contest outcome (${{\rm{\chi }}}_{1}^{2}$ = 5.53; P = 0.018) and period × contest outcome (${{\rm{\chi }}}_{1}^{2}$ = 8.48; P = 0.003) interaction effects. To investigate these interactions in more detail, we ran models separately for each group (i.e. winners and losers). We found that there was neither an age effect (${{\rm{\chi }}}_{1}^{2}$ = 0.40; P = 0.52) nor a period effect (${{\rm{\chi }}}_{1}^{2}$ = 0.21; P = 0.64) on boldness in winners. Contrastingly, boldness in losers decreased with increasing age (${{\rm{\chi }}}_{1}^{2}$ = 12.42; P < 0.001) and increased after the contest (${{\rm{\chi }}}_{1}^{2}$ = 16.33; P < 0.0001) (Fig. 3). Additionally, we detected a moderate but significant adjusted repeatability of boldness in winners, while the estimated repeatability was non-significant in losers (Table 1).

## Discussion

Many studies have investigated the role of morphological traits on contest outcome in animals, but few have looked at the effect of personality on contests. We studied boldness, exploration and activity in Speckled wood (*Pararge aegeria*) butterflies and we subjected males –for which these behavioural variables were quantified– to staged interactions. Boldness decreased with age and butterflies behaved more boldly during the second pre-contest test, whereas exploration and activity were only influenced by ambient conditions. Males which were more explorative and more active before the staged interactions and those with a red wing mark were more likely to win contests. Furthermore, losing a contest caused an increase in boldness, and disrupted the repeatability of this personality trait.

### Repeatability and determinants of behavioural traits

Personality traits are getting increasingly documented in invertebrates, including insects^12^. Here, we show significant repeatability for boldness in a butterfly. Importantly, personality is not necessarily fixed during an individual’s lifespan^37^. Instead, it can change with ontogeny (e.g.^15,38^). Contrary to our expectations, butterflies became shier with increasing age, which is in line with what is observed in a wild population of Blue tits^39^ and in captive Chimpanzees^40^. Several mechanisms may explain this senescence of boldness in our study system: age-related reduction in brain structure size (e.g.^41^), compensation of high levels of boldness in young individuals by other aspects of their phenotype (see^42^), or age-related changes in energetic allocation. Unravelling the precise mechanisms responsible for the senescence of boldness is beyond the scope of this study and would require further testing. In addition to the age-related changes in boldness, we found that the boldness score was higher during the second test. This suggests that butterflies become more familiar with testing conditions and thus habituation *sensu lato* may be taking place^43–45^.

### Drivers of contest outcome in the Speckled wood

Our results confirm that age and morphology have little influence on contest outcome in the Speckled wood^32–34^. Contrary to what has been observed for a sea anemone^22^, boldness was a poor predictor of contest outcome in the Speckled wood and it cannot be considered a RHP trait in this species, at least when contests involve no clear asymmetry in the roles endorsed by contenders. Similar to Rose *et al*.^9^, we found that butterflies with higher exploration/activity scores were more likely to win a contest. This agrees with Kemp and Wiklund^46^ who suggested that intrinsic behaviour may be important to settle contests in the Speckled wood. Active males may have an advantage during contests as they have more opportunities to express offensive behaviours or they may make quicker decisions during contests^21^. Interestingly, we found that only the losers show an age-related decline in boldness, and this both during the pre- and post-contest periods. This suggests that winners and losers not only differ in behaviour, but also in their rate of age-related behavioural change. Note that our study involves naïve individuals that had never experienced territorial contest before. Under natural conditions, males will not only be challenged multiple times by conspecifics during their life, but they will also be rewarded with successful matings. In the Speckled wood butterfly, female encounters increase a male’s motivation to persist in territorial contests and increase the chances of an initial loser to win in subsequent contest^28^. The respective roles of motivation and behavioural traits in setting contest outcomes, and how they interact, remain to be tested.

In addition to the effect of the behavioural traits, we also found that small colour marks affected contest outcome. As we used these marks only to discriminate among males of a pair, this result appears to be an unexpected side effect of our design. The coloured spot was located on the distal part of the forewing and covered less than 3 mm² on average (i.e. about 2% of the forewing’s total surface). As such, it is unlikely to have affected thermoregulation^47^. We found that males with a red wing mark had a higher probability to win a contest compared to green-marked butterflies. This result is surprising because Speckled wood butterflies lack red receptors^48^. Additionally, butterflies would need to be very close to each other to be able to detect these marks. Extrapolating from the visual acuity of the Empress leilia butterfly (*Asterocampa leilia*)^49^, Speckled wood males would need to be at a maximum distance of about 20 cm to be able to detect these marks. Although such proximity is reached during escalated circling contests, this phase typically consists of fast aerial manoeuvres that will likely cause a motion blur^50^ and will decrease the ability to perceive such marks. Consequently, the advantage of red marked individuals (or the disadvantage of green marked) remains puzzling, especially because red and green spots do not naturally occur on wings of the Speckled wood butterfly. Further testing would be needed to elucidate the proximate cause of this difference.

### Winner and loser effects on boldness

Although boldness did not predict contest outcome, fighting reduced the repeatability of this personality trait. The reduction in boldness repeatability was especially strong in losers and losing a contest disrupted the consistency of this trait, similar to what is observed in the sea anemone *A. equina*^51^. Additionally, we found that winners and losers experienced different post-contest changes in boldness (i.e. changes in the mean trait value). In winners, we did not observe any change in boldness between pre- and post-contest periods, whereas boldness increased in losers after losing a territorial contest. This suggests that the magnitude of loser effects on behavioural traits is more pronounced compared to winner effects in the Speckled wood. However, the direction of the loser effect is quite surprising given that losing a contest generally results in decreased boldness^21^ (but see^10^). This could be explained by a process similar to the ‘desperado effect’^52^. When vacant territories become rare and a conventional rule (e.g. size) leaves some individuals unable to gain access to them, high levels of aggression by losers are predicted^52^. After losing a contest, Speckled wood males leave the occupied sunspot, but appear to maintain a perching strategy and re-establish themselves in other, potentially smaller, patches^53^. Because the amount of suitable territories is expected to be limited (at least when the population density is high) and territory holders typically have a higher chance of mating in the Speckled wood^34^, we can expect a desperado effect to develop and to trigger an increase in boldness in losers of territorial contests. Whether such a behavioural change enables losers to take territories in subsequent contests is currently unknown and would require exposing males to multiple contests. As traits determining success in the first *versus* subsequent contests are not necessarily the same^51^, such an experiment would provide insight in the role of boldness in this system.

## Conclusion

In recent years, personality traits have been identified in many animal taxa, but relatively few studies have looked at their implications for territorial contests and more generally for mating strategies^54^. Our results show that winners and losers of contests differ in pre-contest behaviour and, surprisingly, that small artificial ornaments can affect contest outcome. In addition, deterred males experienced an increase in boldness following a defeat. Given that an individual’s mating strategy (i.e. a territorial or satellite/sneaker strategy) has an impact on its reproductive success (e.g.^55–57^), our findings open up interesting research avenues regarding the role of personality traits in territoriality.

## Methods

### Study species and sampling sites

The Speckled wood (*Pararge aegeria* L.) is a common European satyrine butterfly that primarily occurs in woodlands. Caterpillars feed on the leaves of various grass species^58^. In this species, patrolling and perching mate-location strategies are known in males (e.g.^59,60^). In late May and early June 2017, we captured gravid females at five sites in central Belgium. These sites consisted of woodlands (range: 153–1255 ha) with distances between sites varying between 4.7 and 42.3 km. At one site we collected three females, at another site one female, and at the three remaining sites two females each. All these females were brought to the laboratory for oviposition on the grass *Poa pratensis* in individual cages. During oviposition, females could feed *ad libitum* on cotton soaked with a 10% honey solution. As such, we obtained a total of ten families.

### Host plant and butterfly rearing

Host plants were obtained from commercially grown grass turf, later grown on a standardized soil mixture and under standard conditions in a climate room (16 L:8D; 25 °C:16 °C). Throughout the rearing, plants were watered on a regular basis to maintain a moist substrate. From each of the ten families, we randomly selected 24 caterpillars (first or early second instar), which we spread over six pots of host plants. Each pot hence contained four full-sib caterpillars and was enclosed in nylon netting. Caterpillar development took place under identical photoperiod and temperature conditions as for host plant growth. Pots were checked on a regular basis for pupating caterpillars. Pupae were removed from their host plant and placed individually in labelled plastic cups. We checked all cups twice a day for emergence.

### Experimental procedure

Only virgin males were considered for the rest of the experiment. They were left undisturbed until the day following emergence (i.e. age = 1) in order to give enough time for their wings to harden. We obtained a total of 106 males, which were each tested twice for boldness, exploration and activity (see below). Butterflies were first tested at 2.5 ± 1.3 days (mean ± SD; range: 1–6 days), and again at 5.3 ± 1.7 days (mean ± SD; range: 2–10 days) (see Supplementary Table S1 for the precise number of butterflies tested at each age). Tests were conducted between the 8^th^ and 29^th^ of July by one observer (AK) in order to minimize variation in scoring. At the end of each day, all butterflies could feed *ad libitum* from a cotton pad soaked with a 10% honey solution.

### Boldness test

We designed a boldness test inspired by docility tests in birds (e.g.^61,62^) and adapted to our study system. Individual butterflies were placed in a semi-transparent glassine envelope (63 × 97 mm), which allowed the observer to see the butterfly’s movements. We positioned the butterfly (with closed wings) in the centre of the envelope and maintained it in this position by gently pressing two opposite corners of the envelope. We counted the number of struggles during one minute. Here, we define a struggle as a series of leg, head and/or wing movements, interrupted from other such series by pauses of inactivity. As the butterfly is unable to move freely in the envelope, we assume the test mimics a butterfly being stuck in a spider web or held in a bird’s beak. Tests took place in a 25 °C room under constant light conditions. All tested butterflies were then weighed using a microbalance (Ohaus Explorer; accuracy: ± 0.1 mg). One individual was not retested.

### Exploration and activity test

After the boldness test, butterflies were submitted to an exploration/activity test. Each butterfly was released individually at one end of an empty greenhouse tunnel (12 × 4 × 2 m) whose concrete floor was taped to delineate 16 squares of 1.5 × 2 m. The tunnel was installed in a much larger greenhouse maintained at a temperature of 21 °C. Each butterfly was allowed to move freely in the tunnel during four minutes while the observer recorded: (1) the number of squares visited at least once (hereafter: exploration); (2) the number of transitions between squares (hereafter: activity). We additionally recorded temperature (in °C) and light intensity (in lux) during the tests using a data logger (HOBO Pendant Temperature/Light 64 K). All tests were conducted between 11:15 AM and 4:15 PM (UTC + 1).

### Male-male contests and effects on boldness

For a subsample of the males, we staged pairwise contests following a procedure adapted from^33^. Contests took place in a cage (length × width × height: 5.5 × 4.5 × 2.5 m) designed to mimic the essential sensory components of woodland conditions that induce natural male behaviour^63^. This cage was installed in a much larger greenhouse and was covered with a black shade cloth (light-blocking capacity of 90%) from which we removed a circular section (diameter: 1.3 m) to create a sunlit spot tracking the cage floor from 10:30 AM to 4:30 PM (i.e. the period during which contests were conducted). We additionally covered the floor with a woodchips and dry leaves mix and we inserted seven artificial Christmas trees (three of which were moved from time to time to overlap with the sunspot). Contests were conducted only under favourable conditions (i.e. no to low cloud cover) to ensure a clear contrast between the sunspot and the rest of cage during the male-male interactions. Ambient air temperature during the contests was 26.3 ± 1.8 °C (mean ± SD – range: 23.2–30.2 °C).

We established 41 pairs of males by randomly selecting individuals for which boldness, exploration and activity were previously assessed. We avoided testing individuals from the same family against each other, but it happened in six of the contest events due to logistic constraints. We coloured an upperwing spot of each butterfly using a permanent marker (Staedtler permanent Lumocolor, Germany): for one male, this spot was coloured in green; for the other one, in red (see Supplementary Fig. S1). This manipulation aimed at distinguishing males from each other and we were not primarily interested in testing the effect of these colours on contest outcome. The selected spot was located on the distal part of the forewing so as to avoid interference with thermoregulation^47^ and covered a surface of 2.98 ± 0.71 mm² (mean ± SD; N = 25). Butterflies were 8.3 ± 2.6 days old (mean ± SD) at the time of the contest, with the age difference between the two contenders being 2.0 ± 0.7 days (mean ± SD). Butterflies were transported and stored in a cool box at *ca*. 14 °C. Two butterflies were released simultaneously and we observed each pair until dominance was clearly established (usually less than 10 min): butterflies typically made a short initial flight around the cage before trying to settle in the sunspot (i.e. landing either on the floor or on a branch of an artificial tree). When they detected each other, males engaged in an escalated circling phase until dominance was established (see^32^). The dominant male typically chased the subordinate before returning to the sunspot whereas the subordinate tried to escape the enclosure or landed in the shade. On a few occasions we had to stimulate interactions by throwing a small piece of woodchip between the opponents. This caused both males to take flight to chase the moving object and triggered reciprocal detection^33^. We recorded the identity of the dominant male (i.e. the winner) at the end of the observation period and recaptured both butterflies. Within 10 min after the outcome of the contest, both contenders were subjected to a third boldness test, following the procedure described above.

### Morphological measurements

After the last behavioural test, butterflies were killed and stored by freezing (−21 °C). Afterwards, individuals which had been used in contests were placed in a drying oven at 60 °C for 24 h. Total dry body mass was obtained using a microbalance (Ohaus Explorer; accuracy: ± 0.1 mg). Then, butterflies were dissected by carefully separating head, thorax, abdomen, legs and wings. Thoraxes were weighed separately and forewings were scanned (HP F4272) to measure forewing length and forewing area using the image analyser software ImageJ. We retrieved three uncorrelated morphological traits (Pearson’s r < 0.15) which were used as proxies for three types of morphological characteristics: (1) size: forewing area (cm²), (2) wing shape: aspect ratio (4 × forewing length²/forewing area) and (3) relative allocation: relative thorax mass (thorax dry mass/total dry mass).

### Statistical analyses

All statistical analyses were performed with R 3.4.2^64^. We analysed behavioural traits, contest outcome and effects of the latter on boldness using (generalized) linear mixed-effects models (*lme4* and *MASS* packages). Boldness and exploration were modelled with a quasi-Poisson distribution error (log link function), since equivalent models with a Poisson distribution error showed signs of overdispersion. We applied a square-root transformation on activity and we then analysed it with a linear mixed model. All models included age (continuous variable), sequence (categorical variable; first *versus* second trial) and fresh body mass as fixed effects, and population, family and individual identity as random factors. For exploration and activity (which were both measured in the greenhouse tunnel), we additionally included ambient temperature and light intensity as covariates.

Contest outcome was modelled with a binomial distribution error (winner = 1; loser = 0). We included age, forewing area, aspect ratio, thorax ratio and marking colour (red *versus* green) as fixed effects. In order to include behaviour as additional fixed effects, we first conducted a principal component analysis (PCA) on the measured behavioural traits using the *FactoMineR* package. Each repetition was treated as an independent measure of behaviour, resulting in having six behavioural traits used in the PCA. The procedure generated three PCs with eigenvalues greater than one, which were retained for further analyses (Supplementary Table S2). The PC1 correlates positively with exploration and activity; PC2 correlates positively with boldness; and PC3 correlates negatively with exploration during the second trial only. Those three PCs were included as additional fixed effects. Each contest was attributed a single identifier which was included as a random effect.

In order to evaluate the effect of contest outcome (i.e. winning/losing the sunspot) on boldness, we considered the second (i.e. before the male-male contest) and third (i.e. after the contest) boldness measures and we fitted a generalized linear mixed-effects models with a quasi-Poisson distribution error (log link function). Fixed effects included age, status (winner *versus* loser), period (before *versus* after contest) and their two-way interactions. Butterfly identity, family and population of origin were added as random effects. All continuous variables were scaled prior to analysis and we obtained P-values for the fixed effects using Type II Wald tests.

Pearson’s correlations were used to assess the relationships among behavioural traits. The adjusted repeatability of each behavioural trait was estimated using the *rptR* package^65^ and we accounted for overdispersion if appropriate. For boldness, we first estimated repeatability between the first and second trials (i.e. before the contest). We further estimated the repeatability –separately for winner and loser males– for boldness before and after the contest.

## Supplementary information

Supplementary information

## Data Availability

The datasets generated during the current study are available from the corresponding author on reasonable request.

## References

[1] Briffa, M. & Sneddon, L. U. Contest behavior in *Evolutionary behavioral ecology* (eds Westneat, D. F. & Fox, C.) 246–265 (Oxford University Press, 2010).

[2] Parker G (1974). Assessment strategy and the evolution of fighting behaviour. J. Theor. Biol..

[3] Briffa M, Sneddon LU (2007). Physiological constraints on contest behaviour. Funct. Ecol..

[4] Rutte C, Taborsky M, Brinkhof MWG (2006). What sets the odds of winning and losing?. Trends Ecol. Evol..

[5] Trannoy S, Penn J, Lucey K, Popovic D, Kravitz EA (2016). Short and long-lasting behavioral consequences of agonistic encounters between male *Drosophila melanogaster*. Proc. Natl. Acad. Sci..

[6] Kasumovic MM, Elias DO, Sivalinghem S, Mason AC, Andrade MCB (2010). Examination of prior contest experience and the retention of winner and loser effects. Behav. Ecol..

[7] Hsu Y, Wolf LL (1999). The winner and loser effect: integrating multiple experiences. Anim. Behav..

[8] Schuett GW (1997). Body size and agonistic experience affect dominance and mating success in male copperheads. Anim. Behav..

[9] Rose J, Cullen DA, Simpson SJ, Stevenson PA (2017). Born to win or bred to lose: aggressive and submissive behavioural profiles in crickets. Anim. Behav..

[10] Frost AJ, Winrow-Giffen A, Ashley PJ, Sneddon LU (2007). Plasticity in animal personality traits: does prior experience alter the degree of boldness?. Proc. R. Soc. B Biol. Sci..

[11] Réale D, Reader SM, Sol D, McDougall PT, Dingemanse NJ (2007). Integrating animal temperament within ecology and evolution. Biol. Rev..

[12] Kralj-Fišer S, Schuett W (2014). Studying personality variation in invertebrates: why bother?. Anim. Behav..

[13] Bell AM (2007). Future directions in behavioural syndromes research. Proc. R. Soc. B Biol. Sci..

[14] Sih A, Cote J, Evans M, Fogarty S, Pruitt J (2012). Ecological implications of behavioural syndromes. Ecol. Lett..

[15] St-Hilaire É, Réale D, Garant D (2017). Determinants, selection and heritability of docility in wild eastern chipmunks (*Tamias striatus*). Behav. Ecol. Sociobiol..

[16] Rödel HG (2015). Survival costs of fast exploration during juvenile life in a small mammal. Behav. Ecol. Sociobiol..

[17] Santos CD (2015). Personality and morphological traits affect pigeon survival from raptor attacks. Sci. Rep..

[18] Both C, Dingemanse NJ, Drent PJ, Tinbergen JM (2005). Pairs of extreme avian personalities have highest reproductive success. J. Anim. Ecol..

[19] Mutzel A, Dingemanse NJ, Araya-Ajoy YG, Kempenaers B (2013). Parental provisioning behaviour plays a key role in linking personality with reproductive success. Proc. R. Soc. B Biol. Sci..

[20] Vetter SG (2016). Shy is sometimes better: personality and juvenile body mass affect adult reproductive success in wild boars. Sus scrofa. Anim. Behav..

[21] Briffa M, Sneddon LU, Wilson AJ (2015). Animal personality as a cause and consequence of contest behaviour. Biol. Lett..

[22] Rudin FS, Briffa M (2012). Is boldness a resource-holding potential trait? Fighting prowess and changes in startle response in the sea anemone, *Actinia equina*. Proc. R. Soc. B Biol. Sci..

[23] Courtene-Jones W, Briffa M (2014). Boldness and asymmetric contests: role- and outcome-dependent effects of fighting in hermit crabs. Behav. Ecol..

[24] Scott JA (1974). Mate-locating behavior of butterflies. Am. Midl. Nat..

[25] Wiklund, C. Sexual selection and the evolution of butterfly mating systems in *Butterflies: ecology and evolution taking flight* (eds Boggs, C. L., Watt, W. B. & Ehrlich, P. R.) 67–90 (University of Chicago press, 2003).

[26] Kemp DJ, Wiklund C (2001). Fighting without weaponry: a review of male-male contest competition in butterflies. Behav. Ecol. Sociobiol..

[27] Kemp, D. J. Contest behaviour in butterflies: fighting without weapons in *Animal Contests* (eds Hardy, I. C. W. & Briffa, M.) 134–146 (Cambridge University Press, 2013).

[28] Bergman M, Olofsson M, Wiklund C (2010). Contest outcome in a territorial butterfly: the role of motivation. Proc. R. Soc. B Biol. Sci..

[29] Ducatez S, Baguette M (2016). Inter-individual variation in shivering behaviour in the migratory painted lady *Vanessa cardui*. Ecol. Entomol..

[30] Ducatez S (2012). Inter-individual variation in movement: is there a mobility syndrome in the large white butterfly *Pieris brassicae*?. Ecol. Entomol..

[31] Ducatez S, Humeau A, Congretel M, Fréville H, Baguette M (2014). Butterfly species differing in mobility show different structures of dispersal-related syndromes in the same fragmented landscape. Ecography..

[32] Kemp DJ, Wiklund C, Van Dyck H (2006). Contest behaviour in the speckled wood butterfly (*Pararge aegeria*): seasonal phenotypic plasticity and the functional significance of flight performance. Behav. Ecol. Sociobiol..

[33] Kemp DJ, Wiklund C, Gotthard K (2006). Life history effects upon contest behaviour: age as a predictor of territorial contest dynamics in two populations of the Speckled wood butterfly, *Pararge aegeria* L. Ethology.

[34] Bergman M (2007). Mating success of resident versus non-resident males in a territorial butterfly. Proc. R. Soc. B Biol. Sci..

[35] Hulthén K (2017). A predation cost to bold fish in the wild. Sci. Rep..

[36] Wolf M, van Doorn GS, Leimar O, Weissing FJ (2007). Life-history trade-offs favour the evolution of animal personalities. Nature.

[37] Stamps J, Groothuis TGG (2010). The development of animal personality: Relevance, concepts and perspectives. Biol. Rev..

[38] Fisher DN, James A, Rodríguez-Muñoz R, Tregenza T (2015). Behaviour in captivity predicts some aspects of natural behaviour, but not others, in a wild cricket population. Proc. R. Soc. B Biol. Sci..

[39] Class B, Brommer JE (2016). Senescence of personality in a wild bird. Behav. Ecol. Sociobiol..

[40] Massen JJM, Antonides A, Arnold A-MK, Bionda T, Koski SE (2013). A behavioral view on chimpanzee personality: Exploration tendency, persistence, boldness, and tool-orientation measured with group experiments. Am. J. Primatol..

[41] Pasquet A, Toscani C, Anotaux M (2018). Influence of aging on brain and web characteristics of an orb web spider. J. Ethol..

[42] Favati A, Zidar J, Thorpe H, Jensen P, Løvlie H (2016). The ontogeny of personality traits in the red junglefowl. Gallus gallus. Behav. Ecol..

[43] Dingemanse NJ (2012). Variation in personality and behavioural plasticity across four populations of the great tit *Parus major*. J. Anim. Ecol..

[44] Briffa M, Jones N, Macneil C (2016). Responses to threat in a freshwater invader: longitudinal data reveal personality, habituation, and robustness to changing water temperatures in the “killer shrimp” *Dikerogammarus villosus* (Crustacea: Amphipoda). Curr. Zool..

[45] Thys B (2017). The female perspective of personality in a wild songbird: repeatable aggressiveness relates to exploration behaviour. Sci. Rep..

[46] Kemp DJ, Wiklund C (2004). Residency effects in animal contests. Proc. R. Soc. B Biol. Sci..

[47] Wasserthal LT (1975). The role of butterfly wings in regulation of body temperature. J. Insect Physiol..

[48] Briscoe AD, Chittka L (2001). The evolution of color vision in insects. Annu. Rev. Entomol..

[49] Rutowski R (2002). & Warrant, E. Visual field structure in the Empress Leilia, *Asterocampa leilia* (Lepidoptera, Nymphalidae): dimensions and regional variation in acuity. J. Comp. Physiol. A Sensory, Neural, Behav. Physiol..

[50] Land MF (1997). Visual acuity in insects. Annu. Rev. Entomol..

[51] Lane SM, Briffa M (2017). Boldness is for rookies: prefight boldness and fighting success in a sea anemone. Anim. Behav..

[52] Grafen A (1987). The logic of divisively asymmetric contests: respect for ownership and the desperado effect. Anim. Behav..

[53] Bergman M, Wiklund C (2009). Differences in mate location behaviours between residents and nonresidents in a territorial butterfly. Anim. Behav..

[54] Réale, D. & Dingemanse, N. J. Personality and individual social specialisation in *Social Behaviour: Genes, Ecology and Evolution* (eds Szekely, T., Moore, A. J. & Komdeur, J.) 417–441 (Cambridge University Press, 2010).

[55] Takeuchi T (2017). Agonistic display or courtship behavior? A review of contests over mating opportunity in butterflies. J. Ethol..

[56] Kanoh Y (2000). Reproductive success associated with territoriality, sneaking, and grouping in male rose bitterlings, *Rhodeus ocellatus* (Pisces: Cyprinidae). Environ. Biol. Fishes.

[57] Kodric-Brown A (1986). Satellites and sneakers: opportunistic male breeding tactics in pupfish (*Cyprinodon pecosensis*). Behav. Ecol. Sociobiol..

[58] Shreeve TG (1986). Egg-laying by the speckled wood butterfly (*Pararge aegeria*): the role of female behaviour, host plant abundance and temperature. Ecol. Entomol..

[59] Merckx T, Van Dyck H (2005). Mate location behaviour of the butterfly *Pararge aegeria* in woodland and fragmented landscapes. Anim. Behav..

[60] Van Dyck H, Matthysen E (1998). Thermoregulatory differences between phenotypes in the speckled wood butterfly: hot perchers and cold patrollers?. Oecologia.

[61] Brommer JE, Kluen E (2012). Exploring the genetics of nestling personality traits in a wild passerine bird: testing the phenotypic gambit. Ecol. Evol..

[62] Hall ML (2015). Animal personality and pace-of-life syndromes: do fast-exploring fairy-wrens die young?. Front. Ecol. Evol..

[63] Vande Velde L, Van Dyck H (2013). Lipid economy, flight activity and reproductive behaviour in the speckled wood butterfly: on the energetic cost of territory holding. Oikos.

[64] R Core Team. *R: A language and environment for statistical computing*. (R Foundation for Statistical Computing, Vienna, Austria, https://www.R-project.org/, 2018).

[65] Stoffel MA, Nakagawa S, Schielzeth H (2017). rptR: repeatability estimation and variance decomposition by generalized linear mixed-effects models. Methods Ecol. Evol..

