# Supplementary material for: Personality traits influence contest outcome, and vice versa, in a territorial butterfly
Source: Sci Rep. 2019 Feb 26;9:2778. doi: 10.1038/s41598-019-39155-9 (PMC6391398; doi:10.1038/s41598-019-39155-9)
Supplement: Supplementary file 1 — Supplementary information [file 41598_2019_39155_MOESM1_ESM.docx]

Electronic supplementary material

*Scientific Reports*

**Personality traits influence contest outcome, and vice versa, in a territorial butterfly**

Aurélien Kaiser*, Thomas Merckx, Hans Van Dyck

Behavioural Ecology and Conservation Group, Biodiversity Research Centre, Earth and Life Institute, Université catholique de Louvain (UCL), Belgium

*Corresponding author. E-mail: kaiser.aurelien@gmail.com

Figure S1: Pictures of red-marked, green-marked and unmarked wings under normal light (left) and split into their red, green and blue components (converted to greyscale for easier viewing).


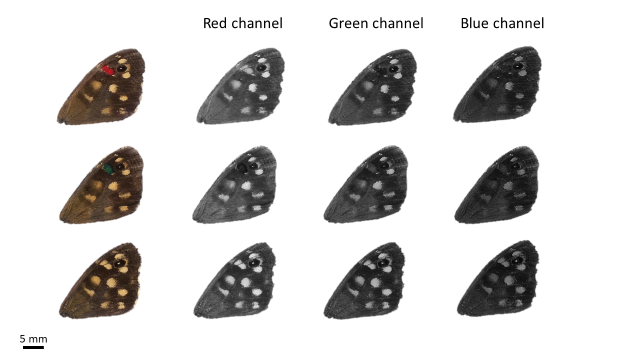


Table S1: Number of tested butterflies at each age for the first and second behavioural trials.

| Age (days) | First trial | Second trial |
| --- | --- | --- |
| 1 | 29 | 0 |
| 2 | 29 | 7 |
| 3 | 16 | 9 |
| 4 | 23 | 16 |
| 5 | 7 | 27 |
| 6 | 1 | 19 |
| 7 | 0 | 18 |
| 8 | 0 | 7 |
| 9 | 0 | 2 |
| 10 | 0 | 1 |

Table S2: Results of the principal component analyses on six behavioural traits (N = 106 individuals). Only PCs with an eigenvalue greater than 1 were retained for the analyses.

|  | PC1 | PC2 | PC3 |
| --- | --- | --- | --- |
| Boldness 1 | 0.212 | 0.703 | 0.389 |
| Boldness 2 | 0.154 | 0.697 | 0.395 |
| Exploration 1 | 0.657 | -0.327 | 0.478 |
| Exploration 2 | 0.618 | 0.191 | -0.584 |
| Activity 1 | 0.632 | -0.472 | 0.379 |
| Activity 2 | 0.676 | 0.203 | -0.499 |
| Eigenvalue | 1.74 | 1.39 | 1.27 |
| Variance explained | 29.01% | 23.18% | 21.19% |
